# Multiplex bisulfite PCR resequencing of clinical FFPE DNA

**DOI:** 10.1186/s13148-015-0067-3

**Published:** 2015-03-17

**Authors:** Darren Korbie, Erica Lin, David Wall, Shalima S Nair, Clare Stirzaker, Sue J Clark, Matt Trau

**Affiliations:** Centre for Personalised Nanomedicine, The University of Queensland, St Lucia, 4072 QLD Australia; Australian Institute for Bioengineering and Nanotechnology, The University of Queensland, Corner College and Cooper Rds (Bldg 75), St Lucia, 4072 QLD Australia; Epigenetics Research Program, Cancer Division, Garvan Institute of Medical Research, 384 Victoria Street, Darlinghurst, NSW 2010 Australia; St Vincent’s Clinical School, Faculty of Medicine, UNSW, St Vincent’s Hospital, 390 Victoria Street, Sydney, NSW Australia; School of Chemistry and Molecular Biosciences, The University of Queensland, St Lucia, 4072 QLD Australia

**Keywords:** Bisulfite PCR, Multiplex PCR, Fluidigm Access Array, DNA methylation, FFPE DNA

## Abstract

**Background:**

The clinical utility of DNA methylation as a predictive or prognostic biomarker requires scalable resequencing protocols for bisulfite-converted DNA. Key features of any validation method should be adaptability for low- or high-throughput needs and high reproducibility, and should only require minimal amounts of precious clinical sample as input material. Critically, this method should also deliver robust results when working with bisulfite-converted DNA extracted from formalin-fixed, paraffin-embedded (FFPE) blocks.

**Results:**

We report here for the first time on comparison studies between the Fluidigm Access Array system and multiplex assays for multiplex bisulfite PCR resequencing. The requirement of the Fluidigm Access Array system for high template amounts and its sensitivity to variations in template quality rendered it unsuitable for bisulfite PCR applications utilizing FFPE DNA. In response to this limitation, we established a multiplex bisulfite PCR assay capable of delivering robust methylation data using minimal amounts of FFPE clinical DNA. To evaluate the parameters and reproducibility of this assay, 57 amplicons were used to prepare sequencing libraries in triplicate for 13 FFPE tumour samples, as well as a series of 5 methylated controls (0%, 25%, 50%, 75%, and 100%). Analysis of this data demonstrated that this multiplex assay had high reproducibility (mean standard deviation of 1.4% for methylation values), was low cost, required low sample input (50 ng of DNA or less), and could be scaled for both low- and high-throughput needs. Notably, ExoSAP-IT (exonuclease I) treatment to remove residual primers in bisulfite resequencing libraries appeared to degrade the library and generate a high-molecular weight smear which may impact on the degree of methylation assessed.

**Conclusions:**

Multiplex bisulfite PCR assays represent a convenient and scalable method for validation and screening of methylated DNA regions from archival FFPE DNA. Moreover, the library construction process detailed here can be rapidly optimized and implemented with a minimal amount of work, can be performed using the standard equipment found in any molecular biology laboratory, and can be easily adapted for use on both genomic DNA and bisulfite DNA applications. However, in preparing bisulfite libraries for sequencing, the use of ExoSAP-IT is not recommended due to potential off-target nuclease effects which may impact downstream methylation analysis.

**Electronic supplementary material:**

The online version of this article (doi:10.1186/s13148-015-0067-3) contains supplementary material, which is available to authorized users.

## Background

The methylation of cytosine at the carbon 5 position (5-methylcytosine) is an epigenetic mark associated with gene regulation [1]. In particular, in mammals, methylation of cytosine residues in the context of cytosine-guanine (CpG) dinucleotides [2,3] is involved in mechanisms of X chromosome inactivation and epigenetic imprinting (that is, the ‘molecular memory’ of genes), as well as the regulation of gene expression in a tissue-specific manner [1,4,5], and aberrant patterns of DNA methylation are associated with developmental pathologies and disease [6]. The role of DNA methylation in cancer has received particular focus over the past 5 years, which is reflected in the decision of the major sequencing initiatives to pursue genome-wide DNA methylation analysis as one of their research goals (for example, The Cancer Genome Atlas [TCGA], [7] and [8], and International Cancer Genome Consortium, [9]).

The focus on DNA methylation is due in part to its excellent potential as a predictive or prognostic biomarker in the aetiology and progression of cancer. To this end, cancer biomarker discovery projects have utilized a spectrum of techniques to generate DNA methylation data which in turn has driven advances in DNA methylation analysis technology, and data from multiple genome-scale methylome projects at single-base-pair resolution is now available [10]. However, the clinical utility of any methylated DNA biomarker requires independent qualitative and quantitative validation, ideally in independent labs using orthologous techniques to those used in the original discovery project. Moreover, key features of any validation method should be scalability (that is, the capacity to be adapted for low-, moderate-, or high-throughput needs), reproducibility, and the requirement for minimal amounts of precious clinical sample as input material.

Here, we report on the assessment of bisulfite resequencing assays for analysis of methylation in clinical formalin-fixed, paraffin-embedded (FFPE) samples. This analysis involved extensive characterization of the Fluidigm Access Array (Fluidigm, South San Francisco, CA, USA) platform and culminated with the development of a separate multiplex bisulfite PCR assay capable of delivering robust methylation data when using FFPE clinical DNA. Key features of the assay are its low cost, low sample input (critical for limited and precious clinical samples), high reproducibility, and scalability for both low- and high-throughput needs. Moreover, the library construction can be performed using the standard equipment found in any molecular biology laboratory, making it accessible for the majority of labs.

## Results and discussion

Recent reports have detailed microfluidic multiplex PCR (MMP-seq) as providing a robust solution for comprehensive, reliable, and high-throughput genetic profiling of clinical tumour samples [11]. MMP-seq utilizes the Fluidigm Access Array system, which employs microfluidic technology to concurrently amplify 48 or 96 samples against an equal number of primer sets. However, while the Fluidigm Access Array platform has been used for somatic mutation screening of clinical FFPE DNA samples, to date, the use of the platform for methylation analysis has only been reported once [12], and its overall efficacy in bisulfite PCR applications utilizing degraded clinical FFPE DNA has not been evaluated. To this end, we embarked on a series of optimization and characterization experiments to assay the overall performance of the Access Array system in high-throughput methylation analysis.

### Assessing fusion primer sequences on bisulfite PCR fidelity

To enable easy preparation of individual amplicon libraries for sequencing, Access Array protocols utilize a fusion primer strategy wherein universal forward and reverse adaptor sequences are added to the 5′ ends of all primers. After the initial gene-specific amplification, sample-specific barcodes and platform-specific sequencing primers (for example, MiSeq or Ion PGM) can then be added using a second round of ‘barcoding’ PCR. However, the inclusion of a 5′ adaptor sequence in bisulfite PCR primers can be problematic due to the relative sequence degeneracy of a bisulfite-converted DNA genome, which leads to extended A or T homopolymer stretches in the sample, resulting in a lack of sequence complexity in PCR primers. The lack of sequence complexity promotes primer self-annealing, leading to off-target effects and PCR ‘dimers’ which can result in a reduction of sequencing depth if the dimers are not effectively removed. As such, although the addition of 20- to 25-bp fusion sequences at the 5′ end of PCR primers simplifies downstream library construction, it also increases the risk of producing dimerization artefacts leading to a decrease in library quality.

To assess the impact of 5′ fusion sequences in bisulfite PCR applications, three different sets of fusion primers were evaluated across seven different genes for their suitability: M13 fusion sequences, fusion primers incorporating Ion Torrent sequencing adaptor sequences, and Fluidigm’s CS fusion sequences. The results from this screen demonstrate that while all primers produced single products under stringent amplification conditions, the M13 and Ion fusion sequences produced pronounced primer dimer products in negative controls (Additional file 1: Figure S1); in comparison, the Fluidigm CS fusion sequences did not generate dimer products in any of the screening reactions, which suggests that in situations where the template was limiting, the CS sequences may perform better. Based on this result, the CS1 and CS2 sequences were selected for further use, and all future experiments employed CS fusion primers.

### Multiplex pre-amplification of bisulfite-converted cell line DNA

The Access Array system recommends using 50 ng of DNA as the template input amount, in a total volume of 5 μl; assuming that 1 ng of DNA is equivalent to 333 copies, this equals approximately 100 copies of DNA in one Fluidigm nanowell (refer to ‘Methods’ for these calculations). Given DNA degradation due to FFPE fixation and extraction, together with potential inaccuracies in DNA quantitation, it was therefore considered likely that the number of PCR-amplifiable copies of DNA present would be considerably less than 100 if the recommended DNA input amount was followed. Limited DNA in the initial bisulfite reaction can result in substantial PCR bias [13] particularly if heterogeneous DNA methylation is present in a sample (for example, due to variable tumour load within a sample). Although increasing the total amount of DNA template input represents one possible solution when confronted with a limiting amount of DNA copies, only 28% of input material ever participates in amplification on an Access Array chip nanowell (refer to ‘Methods’ for this calculation); as such, increasing DNA input amount would always sacrifice 72% of the total sample, which is an untenable alternative when working with limited clinical DNA. For these reasons, pre-amplification protocols were assessed for their performance on bisulfite-converted DNA, as a way to leverage greater utility out of limited sample input.

To assess pre-amplification as a strategy for working with bisulfite-converted DNA, individual primer pairs were first screened against control cell line DNA. Only amplicons that produced clear distinct products with no observable secondary bands were selected for use in pre-amplification, and based on this, 48 primer pairs were identified. Non-overlapping primer pairs were then pooled into three combinations: eight different 8-plex pools, two 24-plex pools, and one 48-plex pool, after which each multiplex pool underwent 15 rounds of PCR. This pre-amplification PCR was followed by ExoSap-IT treatment to remove residual primers, in an attempt to increase the fidelity of downstream singleplex PCR by removing interfering oligos.

**Figure 1:**
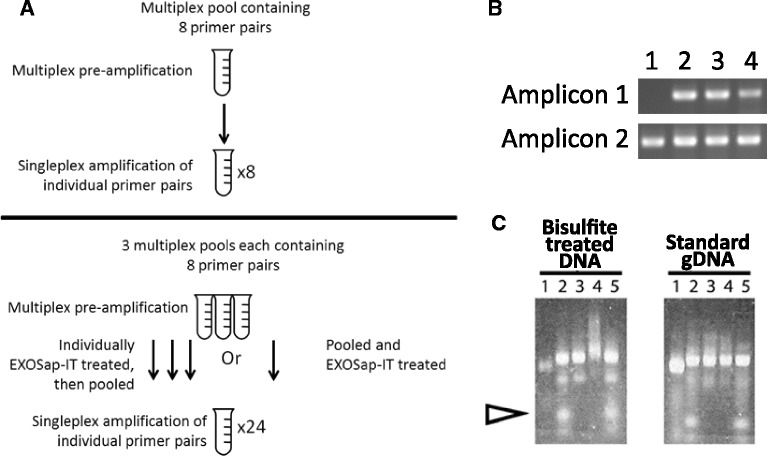
**Pre-amplification multiplex results for bisulfite DNA samples. (A)** A flow diagram outlining the different conditions examined with respect to multiplexability, pooling, and exonuclease treatment. **(B)** The results of different conditions after 15 cycles of multiplex pre-amplification. Forty-eight primer pairs were assessed for the multiplexability in a pre-amplification reaction. All samples had the same amount of input DNA. **Lane 1**: A positive control involving singleplex PCR reaction of an individual primer pair, with the same DNA template amount as used in the pre-amplification. **Lane 2**: Eight-plex pre-amplification reaction and ExoSAP-IT treatment of individual pre-amp reactions, followed by pooling and singleplex amplification (as illustrated in the upper panel of A). **Lane 3**: Results of three different 8-plex reactions pooled together first then ExoSAP-IT treatment of the combined pool, for a total of 24 amplicons in the ExoSAP-IT treatment, followed by singleplex amplification of a primer pair (as illustrated in the lower panel of A). **Lane 4**: Twenty-four-plex pre-amplification results. **(C)** The effect of ExoSAP-IT treatment on bisulfite libraries, as compared to gDNA amplicons. The arrow indicates where primers migrate on the gel. **Lane 1**: Pool of 48 amplicons prior to barcoding PCR. **Lane 2**: Sample library after barcoding PCR. Primers are visible at the bottom of the lane. **Lane 3**: Sample library after ExoSAP-IT treatment at 37°C. **Lane 4**: Sample library after ExoSAP-IT treatment at 37°C, followed by heat inactivation of the ExoSAP-IT at 80°C. Note the higher molecular weight smear in the methylation library, which is not observed with gDNA amplicons. **Lane 5**: Sample library cycled at 37°C, followed by 80°C heat denaturation step, but with no ExoSAP-IT.

Based on the pre-amplification pools and ExoSAP-IT parameters outlined above, four different pre-amplification screening conditions were assessed, as outlined in Figure 1A:

1. Eight-plex pre-amplification pools wherein each 8-plex pool was treated with ExoSAP-IT individually to remove residual primers after pre-amplification, prior to second-round singleplex PCR
2. The effect of first pooling three different 8-plex reactions together (for a total of 24 amplicons), followed by ExoSAP-treatment of the combined 24-pool, then individually amplifying
3. A combined 24-plex pre-amplification, followed by second-round singleplex PCR
4. A combined 48-plex pre-amplification, followed by second-round singleplex PCR

Singleplex reactions were run with each primer pair as a positive control, and representative results are shown in Figure 1B. Preliminary results with cell line DNA demonstrated that the pre-amplification successfully increased the number of available copies in second-round PCR (Figure 1B, Amplicon 1, lanes 1 and 2), and up to 24 primer pairs could be pooled together and then amplified individually with no observable dimer effects. However, 48-plex pre-amplification resulted in substantial dimer product after second-round singleplex PCR (data not shown), and for this reason, 24-plex amplification was selected as the ideal condition.

The pre-amplification pool was then used to individually amplify each of the 48 targeted regions on a standard thermocycler, after which an aliquot of each amplicon was pooled together to construct the sequencing library. Prior to the final barcoding PCR (wherein sequencing adaptors and sample specific barcodes are added), ExoSAP-IT was again used to remove residual gene-specific primers. This step was required because many amplicons were partially overlapping, and if residual primers are not removed, smaller subproducts could be generated leading to a sequencing library dominated by small amplicon species (data not shown). However, while ExoSAP-IT successfully removed residual primers, exonuclease treatment of bisulfite resequencing libraries appeared to generate a smear of high-molecular weight products not present in the original sample, whereas a similar effect was not observed using genomic DNA (gDNA) amplicons (Figure 1C). This effect was reproducibly observed only with bisulfite libraries and only when the heat denaturation step to inactivate the ExoI/SAP enzymes was included; when the heat inactivation step was omitted, no high-molecular weight smears are observed. Moreover, this is not attributable to exposure to heat, as thermal cycling of the sample library in the absence of ExoSAP-IT does not produce high-molecular smearing of the methylation library (Figure 1C). Although the smeared high-molecular weight product could be used for sequencing (data not shown), due to concerns that off-target nuclease effects could potentially introduce bias towards or against methylated sequences, the use of ExoSAP-IT was discontinued and Agencourt XP beads (Beckman Coulter, Inc., Fullerton, CA, USA) were used to remove residual primers instead.

### Multiplex pre-amplification of bisulfite-converted FFPE clinical DNA

**Figure 2:**
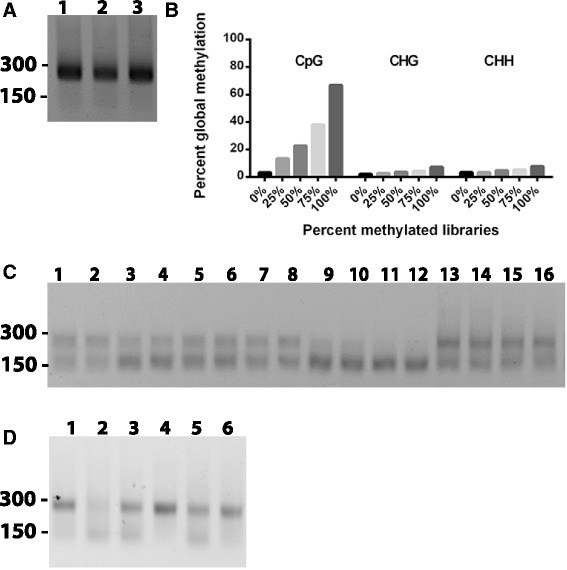
**Bisulfite libraries prepared using the Fluidigm Access Array system. (A)** Low-throughput libraries prepared manually were observed to produce strong dominant bands of the expected size with minimal visible dimer product, when visualized by agarose gel. Size in base pairs is indicated to the left **(B)** Sequencing results for the percent global methylation of the control libraries prepared manually. **(C)** Preliminary results with the Fluidigm Access Array platform resulted in weakly amplifying sequencing libraries with prominent dimer products. **(D)** After extensive optimization to identify the critical parameters, pre-amplification under ideal conditions still gave variable library performance using the Access Array system, with minor differences in pre-amplification primer concentration or the number of cycles of pre-amplification leading to failed libraries (that is, lane 1 vs lane 2). **Lane 1**, 200 nM primer, 15 cycles pre-amplification, GoTaq Flexi buffer; **lane 2**, 50 nM primer, 15 cycles pre-amplification, GoTaq Flexi buffer; **lane 3**, 200 nM primer, 15 cycles pre-amplification, Roche HF buffer; **lane 4**, 200 nM primer, 20 cycles pre-amplification, GoTaq Flexi buffer; **lane 5**, 50 nM primer, 20 cycles pre-amplification, GoTaq Flexi buffer; **lane 6**, 200 nM primer, 20 cycles pre-amplification, Roche HF buffer.

The above control experiments with high-quality cell line DNA indicated good overall performance of the bisulfite PCR pre-amplification assay in producing a final library, and therefore the utility of the assay using clinical FFPE DNA was then assessed. To this end, 13 FFPE breast tumour samples, as well as a series of 5 methylated controls (0%, 25%, 50%, 75%, and 100%), were evaluated in the pre-amplification strategy outlined above, which involved 44 well-performing primer pairs divided between two 20-plex and 24-plex pre-amplification pools which underwent 15 cycles of pre-amplification. An aliquot of this mix was then used in 44 individual singleplex PCR reactions (one for each primer pair) for another 35 cycles, using a standard PCR thermocycler. After checking all products by gel, an aliquot of each amplicon was pooled together followed by 15 rounds of barcoding PCR amplification and finally gel purification; these libraries were observed to give prominent single bands with no visible dimer product (Figure 2A) and were successfully sequenced. Sequenced libraries demonstrated high mappability (that is, over 90% of reads mapped to the reference index); however, it was noted that the methylated controls included in the analysis were below the methylation percentages expected, suggesting potential bias towards unmethylated transcripts (Figure 2B), possibly as a result the total number of cycles used (15 pre-amp + 35 secondary + 15 barcoding = 60 cycles of amplification).

The above experiments demonstrated that the pre-amplification process was working successfully, and therefore to assess the performance of the Access Array platform, the same pre-amplification samples previously used were directly loaded onto a 48 × 48 Access Array microfluidic chip and subjected to the same amplification, purification, and barcoding parameters. However, libraries prepared using the Access Array chip were all noted to produce strong dimer products, with many samples showing no library present at the expected size (Figure 2C). Despite repeated attempts, no high-quality libraries were generated from these samples using the Access Array system, in contrast to the prior success in manually amplifying up individual primer pairs (Figure 2A).

Given the weak performance of the Access Array chip compared to low-throughput methods, extensive optimization experiments were then conducted to determine the potential factors that could impact overall amplification fidelity and yield when working with bisulfite resequencing libraries. As the presence of dimer products in the preliminary optimization experiments was typically only observed in negative control experiments (Additional file 1: Figure S1), it was concluded that poor performance of the Access Array system could be due to insufficient template copies being present in the nanofluidic chambers, perhaps as a result of poor DNA template quality due to FFPE degradation and bisulfite conversion. As such, it was hypothesized that increasing the number of copies generated during the pre-amplification step could potentially improve Access Array performance.

To identify the critical pre-amplification parameters necessary when using the Access Array for bisulfite PCR, an extensive series of optimization experiments was conducted: 24 different pre-amp conditions were evaluated which investigated the effect of varying the number of PCR cycles (15 to 30 cycles), annealing temperature (55°C to 72°C), primer concentration (25 to 200 nM), MgCl_2_ concentration (1.5 to 7.5 mM), different final concentrations of Taq buffer (0.25× to 2×), different PCR buffers (GoTaq Flexi [Promega, Madison, WI, USA], Roche High Fidelity [Roche Life Science, Indianapolis, IN, USA]), cycling conditions (touch-up, touch-down, and standard PCR amplification), and enhancer formulations and concentrations (DMSO, betaine, BSA, DTT, formamide). Despite extensive optimizations to identify the critical parameters in pre-amplification, performance with the Access Array platform was still variable and it was observed that subtle differences in the number of cycles and total final pre-amplification primer concentration could dramatically affect final library results (that is, 50 vs 200 nM primer pool or 15 vs 20 cycles of pre-amplification PCR; Figure 2D).

### Optimization of custom bisulfite PCR multiplex assays

Initial experiments with 24-plex pre-amplification bisulfite pools had demonstrated that pre-amplification of bisulfite libraries could be used to increase the number of available copies of the template (Figure 1B, lanes 1 and 4). Based on this observation, it was hypothesized that if conditions were sufficiently optimized, it should be possible to take the pre-amplification pool template and directly perform the barcoding PCR step, without an intermediate second-stage amplification on the Access Array chip.

Initial optimization experiments were conducted with gDNA amplicons. gDNA primers were run in real-time quantitative PCR (qPCR) under standard conditions, and two different final concentrations of primers were assessed (300 vs 100 nM). Under these conditions, Cts were observed to range from 18.2 to 38.5, and based on the results, primer pairs with proximal Ct values were pooled together, taking care to ensure that primers for overlapping regions were separated. This gave seven pools containing five primer pairs each, after which each pool was then amplified at final concentrations of 1,500, 500, 300, and 100 nM to assess the effect to of primer concentration on amplicon proportionality in the final library. After amplification, each pool concentration was combined and sequenced on both MiSeq and Ion Torrent platforms to compare reproducibility of representation. Amplicon representation across the four samples was normalized per pool and the proportion of each amplicon in the final sample assessed (Additional file 2: Figure S2b); the highest primer concentrations were observed to give the most normalized coverage, with increasing variation in amplicon counts as final primer concentration became more dilute, an effect which was independent of sequencing platform. However, while higher primer concentrations led to a more normalized coverage, they were also observed to generate more dimer products (data not shown).

**Figure 3:**
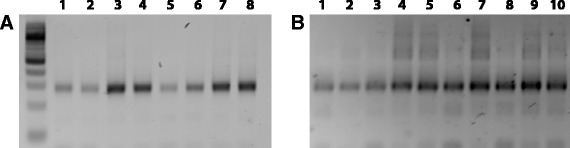
**Representative libraries prepared using the custom bisulfite PCR multiplex assay.** After optimizing primer concentration for the individual primer pairs as well as the overall pools, barcoding of the final libraries demonstrated that the assay performed well on both high-quality white blood cell DNA **(A)** and degraded clinical FFPE samples **(B)**. In comparison, even substantially degraded FFPE DNA which completely failed in Fluidigm Access Array (lanes 8 to 10 in Figure 2C) performed well with this multiplex assay (panel B lanes 1 to 3).

To determine which bisulfite PCR primer pairs should be pooled together, a similar strategy was employed. In brief, real-time qPCR was performed for 84 different amplicons; Cts ranged from 25.5 to 37.1. Based on these results, primer pairs with proximal Ct values were pooled together, taking care to ensure that primers for overlapping regions were separated, which resulted in 59 amplicons spread across 8 pools (five pools of 8, two pools of 6, and one pool of 5). Initial screening of the pools evaluated different final primer pool concentrations (from 1 μM final to 125 nM final) as well as different cycling parameters, enhancers, and MgCl_2_ concentrations; two primer pairs were excluded due to their tendency to cause dimers. After optimizing primer concentration for both the individual primer pairs as well as the overall pools, the same 13 clinical FFPE samples previously used on the Fluidigm platform were subjected to the custom bisulfite multiplex assay. Barcoding of the final libraries demonstrated that all eight pools were giving high-quality libraries (Figure 3A,B) and even substantially degraded FFPE DNA which completely failed in Fluidigm Access Array performed robustly (lanes 8 to 10 in Figure 3B, as compared to Fluidigm libraries in Figure 2C). These clinical samples were also sequenced using the Illumina MiSeq (Illumina, San Diego, CA) to determine the proportionality of amplicon representation and overall methylation state; libraries for each clinical sample were prepared three times on different days to assess reproducibility, and methylated control samples were also included.

**Figure 4:**
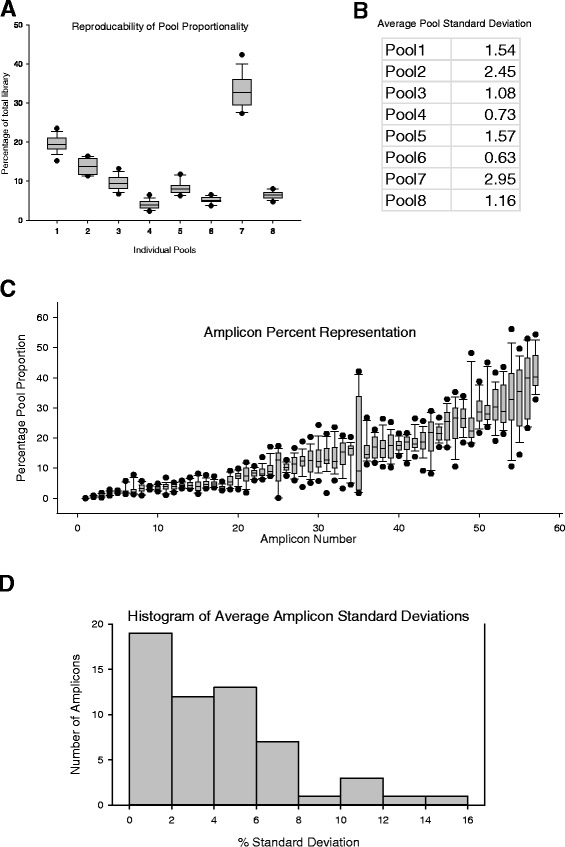
**Sequencing results.** Sequencing results for libraries prepared in triplicate for 13 FFPE tumour samples, as well as a series of 5 methylated controls (0%, 25%, 50%, 75%, and 100%) using the custom bisulfite PCR multiplex assay. **(A)** The proportions of each pool across 54 libraries, determined as a total amount of each library. ***Y-axis***: *Percentage of total library* = *Total number of reads for a pool* ÷ *Total number of reads for the library*. Although pool 7 was observed to dominate all libraries, the proportion of each pool across 54 samples was maintained at consistent levels. Whiskers: 10th to 90th percentiles; black circles: 5th and 95th percentiles. **(B)** The average standard deviation in 8-plex pool proportions observed across all libraries. *Average pool standard deviation* = *(The sum of all standard deviations for a single pool* ÷ *The total number of entries, that is, the mean value)*. Across 13 FFPE samples amplified in triplicate, pool proportionality was maintained within similar values across all samples and libraries. **(C)** The proportion of each amplicon in each of the libraries, calculated as a percentage of its original 8-plex pool. *Percentage pool proportion* = *Total number of reads for an amplicon* ÷ *Total number of reads for its pool*. Whiskers: 10th to 90th percentiles; black circles: 5th and 95th percentiles. **(D)** Histogram showing the distribution of the average standard deviations for all 57 amplicons in the assay. Across 13 FFPE samples amplified in triplicate, the proportion of each of the 56 amplicons was maintained at consistent levels. *Average standard deviation* = *The sum of all standard deviation values for a single amplicon (as a percentage of its original 8-plex pool as outlined in C)* ÷ *The total number of entries*.

Of the 57 amplicons assayed, only one amplicon was observed to consistently fail across all samples, and the remaining 56 amplicons were all present in the assay. Of the 56 amplicons remaining, the total reads for most amplicons were typically observed to be within an order of magnitude of each other, with the difference in total read numbers between the amplicons with the lowest and highest counts typically showing no more than two orders of magnitude difference (Additional file 3: Figure S3). The variability and reproducibility in the proportions of each pool as a total amount of each library were also determined, and the amount of each pool as a percentage of each library was calculated. Across 13 samples amplified in triplicate, pool proportionality was maintained within similar values across all samples and libraries (Figure 4A), with standard deviations of pool proportions less than 3% (Figure 4B). Although pool 7 was observed to dominate all sequencing libraries, subsequent experiments using a reduced concentration of primers for pool 7 reduced the amount present to levels proportional with the other pools (data not shown).

To assess reproducibility of amplicon coverage, the representation of each amplicon across the 54 libraries was determined, after normalizing the total number of reads for an amplicon based on its pool of origin. This analysis showed that while there was a broad distribution of reads/amplicons (Figure 4C), each amplicon was maintained at moderately consistent values across all 54 samples, with most amplicons exhibiting less than 8% variability in total read numbers between samples and replicates (Figure 4C).

### Reproducibility of methylation levels across a region of interest

**Figure 5:**
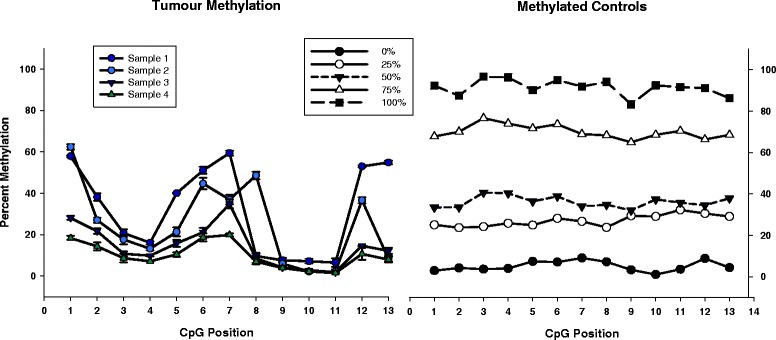
**Representative methylation results.** Representative data of 4 out of the 13 clinical FFPE samples assayed are shown. FFPE breast tumour samples were assayed in triplicate, along with a set of methylation controls (0%, 25%, 50%, 75%, and 100% methylation controls). Whereas the methylation values for the control samples were observed to be maintained at consistent levels across the region of interest, the breast cancer samples were observed to give unique patterns of methylation.

To assess assay reproducibility in determining the methylation state, 293 CpGs were examined in triplicate across a separate set of 13 different tumour samples. Overall assay performance based on methylated controls and clinical FFPE tumour samples indicated consistent assay performance. A representative region along with methylated controls is shown in Figure 5 for 13 CpGs; a larger bias plot showing representative data across 68 CpGs is also shown in Additional file 4: Figure S4. The lowest SD observed across 293 CpGs was 0.0043%, with a mean SD of 1.5%. The maximum SD was calculated to be 23.8%; however, this high value was due to one erratic amplicon which gave consistently high SDs across its length, and after this amplicon was removed, the maximal SD observed across 281 CpGs dropped to 8.04% (Additional file 5: Table S1). Moreover, although standard deviation values are present in Figure 5, they were observed to be so small that the standard deviation spread is not readily visible, and for this reason, the values have been included in a separate table (Additional file 6: Table S2).

## Conclusions

There is a requisite need for scalable and cost-effective resequencing strategies, particularly as relates to methylation analysis of clinical FFPE samples by bisulfite PCR. For example, the recent explosion of data from the TCGA with respect to cancer methylated biomarkers first requires validation by independent labs before they can be confidently translated to a clinical setting. To address this need, we have embarked on a comprehensive set of optimization experiments focused on a scalable resequencing protocol for bisulfite PCR applications. Our analysis involved a highly detailed deconstruction of the key parameters required for effective pre-amplification of bisulfite libraries on the Fluidigm Access Array system and concluded with a multiplex PCR protocol which is effective for use with both gDNA and bisulfite DNA samples. Two key features of this assay are the ability to generate high-quality resequencing libraries using degraded FFPE samples and the ability to use subnanogram amounts of clinical DNA per amplicon once sufficient multiplexability is reached.

An alternate platform for high-throughput resequencing is the Fluidigm Access Array system, which employs nanofluidics for high-throughput PCR amplification and enables analysis of multiple amplicons across 96 samples simultaneously. While this platform has been used for somatic mutation screening of clinical FFPE DNA, at present, there is only one report in the literature on its use for bisulfite PCR applications [12], and to date, no assessment has been performed on its utility when using bisulfite-converted clinical FFPE DNA samples. To evaluate the effectiveness of this platform for high-throughput methylation analysis, we therefore embarked on a comprehensive series of bisulfite PCR optimization experiments. In conducting these optimization experiments, we concluded that the small nanoliter size of the reaction chamber in an Access Array chip made the assay highly sensitive to the number of copies present for amplification; following the recommended Fluidigm protocol would deliver approximately 100 copies of template per well, in the absence of pre-amplification. However, in instances where the sample DNA quantitation is less than completely accurate, and/or when the sample may be partially degraded due to FFPE fixation and extraction, this recommendation would almost certainly result in fewer than 100 copies of DNA being analyzed, which risks introducing substantial bias in the methylation analysis. For this reason, we attempted to employ a pre-amplification strategy when working with our clinical FFPE samples on the Access Array system. In doing so, one recurring theme in optimizing the Access Array for use in bisulfite PCR was the sensitivity of the platform to minor differences in pre-amplification parameters. Our extensive optimization experiments indicated that a template of moderately high quality would require approximately 20 cycles of multiplex pre-amplification, another 35 cycles of singleplex amplification on the Fluidigm Access Array, and finally an additional 15 cycles of barcoding PCR, for a total of 70 cycles of amplification. However, it is generally accepted that when generating NGS libraries, total amplification cycles should be kept to an absolute minimum, and this is considered even more critical when working with bisulfite PCR as a high number of PCR cycles can skew methylation percentages [14]. Although it was possible to slightly reduce the total number of cycles, subtle differences in amplification parameters (that is, 50 vs 200 nM primer pool, 15 vs 20 cycles of pre-amp PCR) were observed to completely alter the success and failure of bisulfite libraries on the Fluidigm system. This suggests a sensitivity to template quality and input which can be difficult to guarantee when working with degraded FFPE samples, even after pre-amplification parameters have been empirically optimized. Although one option could be to increase the total DNA input amount (that is, from 50 to 150 ng), we considered this an unideal option, as clinical DNA is limiting and only the minimum amount necessary should be used. As such, while the recommended pre-amplification parameters for the Access Array may be effective for genomic DNA, they were insufficient when working with bisulfite-converted clinical FFPE samples.

In response to these challenges, we developed a multiplex PCR assay for bisulfite libraries which uses the same recommended template input amount as the Fluidigm Access Array system (50 ng), but which offers higher sensitivity, cheaper costs, and faster turnaround times. While the Fluidigm system can potentially offer greater sample throughput based on multiplexing the primer input nanowells, in our experience, this has led to substantial dimer products in the final library. Moreover, successful library construction ultimately required over 70 rounds of PCR amplification, which risked introducing artefact and bias into methylation analysis [14]. In contrast, our optimized protocol requires only 30 rounds of amplification from start to finish and is suitable for both genomic and bisulfite-converted DNA applications. Moreover, the lowest SD observed across the entire assay was 0.0043%, with a mean SD across all 293 CpGs of only 1.5%. Although we did not perform a comparison of methylation values between tumour samples and normal controls in this manuscript, we believe the high reproducibility of our assay will support robust statistical comparisons between healthy and pathogenic samples and/or fixed and unfixed samples.

To date, this assay has been performed over 200 times on FFPE DNA samples with excellent success, and we are confident that improvements to the assay will continue to increase both the throughput and sensitivity, as well as reduce the total amplification cycles needed.

## Methods

### Fusion primer sequences

Fusion primer sequences used were as follows: M13F, GTAAAACGACGGCCAG; M13R, CAGGAAACAGCTATGAC; Ion P, CCTCTCTATGGGCAGTCGGTGAT; Ion A1, CTGCTGTACGGCCAAGGCG; CS1, ACACTGACGACATGGTTCTACA; and CS2, TACGGTAGCAGAGACTTGGTCT.

Bisulfite DNA conversions were performed using either manual protocols [15] or commercial kits (MethylEasy Xceed P/N ME002, Human Genetic Signatures, North Ryde, NSW, Australia), as per the manufacturer’s protocols. For each conversion, DNA was first quantified with the Qubit dsDNA BR Assay Kit (Life Technologies, Grand Island, NY, USA), and based on the available sample material, between 100 ng and 1 μg of material was bisulfite converted at a time. Conversion took place at 80°C for 45 min, followed by resuspension in low TE (10 mM Tris-Cl, pH 8.0, 0.1 mM EDTA).

### Bisulfite PCR conditions

A PCR Master Mix recipe for bisulfite-converted DNA reagents was made using a 100-μl standard reaction using the following recipe. Promega Hot Start GoTaq with Flexi buffer (M5005) was used. The final PCR reaction had the following components at the indicated concentrations: 5× green (1×), CES 5×, (0.5×, N.B. refer to [16] for CES recipe), MgCl_2_ (4.5 mM), dNTPs (200 μM each), primers (forward and reverse at 100 mM), Hot Start Taq (0.025 U/μl), DNA (2 ng/μl). Amplification took place on either an Eppendorf ProS 96 well or an Eppendorf Pro 384 well thermocycler (Eppendorf, Hamburg, Germany). Cycling conditions were as follows: 94°C, 5 min; 12 cycles (95°C, 20 s; 60°C, 1 min); 12 cycles (94°C, 20 s; 65°C, 1 min 30 s); and 65°C, 3 min, 10°C hold. PCR products were evaluated using standard agarose gel electrophoresis techniques with SB buffer. ExoSAP-IT (P/N 78201 1 ML, Affymetrix, Santa Clara, CA, USA) was used to remove residual primer leftovers in the PCR, and the reaction was carried out according to the manufacturer’s protocol.

### Barcoding PCR conditions

In a 100-μl reaction, the following were the final concentrations: 1× GoTaq Green Flexi buffer; 0.25× CES; 4.5 mM MgCl_2_; 200 μM dNTPs; 0.05 U/μl Hot Start Taq; 25 μl of pooled template after Agencourt XP bead cleanup; and 20 μl MiSeq (Fluidigm PN FLD-100-3771) or Ion Torrent (200 nM). Amplification took place on either an Eppendorf ProS 96 well or Eppendorf Pro 384 well thermocycler. Cycling conditions were as follows: 94°C, 5 min; 9 cycles (97°C, 15 s; 60°C, 30 s; 72°C, 2 min); 72°C, 2 min; and 6°C, 5 min. The Access Array system was run according to the manufacturer’s recommended protocols.

### Copy number calculations

1 bp dsDNA = 615 g/mol. 1 human genome = 3e^9^ dsDNA bases. ((615 g/mol/dsDNA base) × (3e^9^ dsDNA bases))/6.022e^23^ = 3.075 pg/human genome.

### Fluidigm copy number/well calculations

50 ng × 333 DNA copies/ng of DNA = 16,650 DNA copies; 16,650 copies ÷ 5,000 nl = 3.33 copies/nl; (30 nl Access Array well) × (3.33 copies/nl) = 100 copies/nanowell.

### Fluidigm Access Array volume calculations

48 nanowells × 30 nl = 1,440 nl; 1,440 nl/5,000 nl total volume = 28.8% volume which participates in amplification.

### Cells and DNA extractions

MCF7 cells were used for control cell line DNA. DMEM supplemented with 10% FBS, 10 μg/ml insulin, 1 mM sodium pyruvate, and 1× antibiotic/antimycotic was used. DNA extractions from cell line DNA utilized the DNeasy Blood and Tissue Kit from Qiagen (Venlo, The Netherlands), according to the manufacturer’s protocol. Clinical samples were extracted from FFPE blocks using the PAXgene Tissue DNA Kit (PN 767134, Qiagen) according to the manufacturer’s instructions. Five 8-μm sections were processed at a time for each tissue block, although the amount of tissue material for each specimen varied.

### Methylated controls

One hundred percent methylated DNA was commercially sourced from NEB (CpG Methylated Jurkat Genomic DNA, Cat# N4002S). Zero percent methylated DNA was created by performing whole-genome amplification of commercially bought human genomic DNA (Roche Human Genomic DNA Cat# 11691112001), using the Qiagen REPLI-g whole-genome amplification kit, according to the manufacturer’s instruction. Graduated methylated controls (that is, 25%, 50%, 75%) were made by determining the amount of amplifiable DNA in the 100% and 0% methylated DNA samples using qPCR and pooling 0% and 100% methylated DNA in the proportions needed to produce the final methylated control.

Sequencing was performed on either a MiSeq or Ion Torrent sequencer. MiSeq runs used the MiSeq Reagent Kit v2 (300 cycles; PN MS-102-2002). PGM runs used either the OT2 200 bp and 200 bp Ion PGM Sequencing Kit (Life Technologies) with a 314 chip or the OT2 400 bp kit and the 400 bp Ion PGM Sequencing Kit with a 314v2 chip. MiSeq sequencing utilized custom sequencing adaptors, as described in the Fluidigm Access Array manual.

### Bioinformatics

Adaptor trimming employed *Trim galore* (options: --length 100). Mapping used the *Bismark* methylation mapping program [17] running *Bowtie2* [18] (options: --bowtie2 -N 1 -L 15 --bam -p 2 --score L,-0.6,-0.6 --non_directional; bismark_methylation_extractor -s -merge_non_CpG –comprehensive --cytosine_report). To reduce computational overhead, mapping took place against only those genomic regions which were being investigated, plus an additional 100 bp to 1 kb of flanking sequence. Graphing and analysis employed SigmaPlot 12.5 and Excel 2010.

## Additional files

Additional file 1: Figure S1. To assess the performance of different fusion sequences in targeted resequencing, seven different pairs of bisulfite PCR primers were evaluated with and without additional sequences added to their 5' end. (−) = No template control; Std = primers with no fusion sequence; M13 = primers with M13 sequences; Ion = primers with Ion Torrent P and A1 sequences; Fld = Fluidigm’s CS sequences. Although all primer sets performed well under stringent conditions, only Fluidigm’s CS sequences gave no observable dimer product in the negative control. Additional file 2: Figure S2. Thirty gDNA primer pairs were divided equally into six pools and amplified concurrently at four different primer concentrations. Each pool was then sequenced on either an Ion Torrent (A) or MiSeq (B) to assess individual amplicon proportion, as well as whether particular sequencing platforms alter amplicon proportionality. The *Y*-axis represents the percentage proportion for each amplicon, as a total of its particular multiplex pool. Whiskers: 10th to 90th percentiles; black circles: outliers. Additional file 3: Figure S3. The distribution of read counts for 56 bisulfite DNA amplicons across 54 libraries. Although the spread in read numbers between the lowest and highest amplicons for each library is less than two orders of magnitude (that is, 200 to 20,000), the majority of amplicons have read counts which cluster within one order of magnitude of each other. Whiskers: 10th to 90th percentiles; black circles: 5th and 95th percentiles. Additional file 4: Figure S4. Representative methylation bias plot for 68 CpGs across 8 amplicons in the library. Although particular CpG positions are observed to deviate from the median value (for example, CpG 62 and 64), overall, the assay is able to distinguish between different methylation percentages across every CpG. Additional file 5: Table S1. Spread of standard deviation methylation values across Watson strand amplicons, based on three technical replicates. The maximal standard deviation observed in this dataset was due to a single amplicon at low coverage; removing this amplicon reduced the maximum standard deviation observed for a single CpG from 23.8% to 8.04%. Additional file 6: Table S2. Standard deviation values for tumour samples 1 to 13 across three technical replicates for one representative amplicon (N.B. Only samples 1 to 4 are shown in Figure 5). The standard deviation represents the value by which the technical replicates for each sample deviate from the mean percent methylation value.

## References

[1] Jones PA (2012). Functions of DNA methylation: islands, start sites, gene bodies and beyond. Nat Rev Genet.

[2] Holliday R, Pugh JE (1975). DNA modification mechanisms and gene activity during development. Science.

[3] Riggs AD (1975). X inactivation, differentiation, and DNA methylation. Cytogenet Cell Genet.

[4] Wutz A (2011). Gene silencing in X-chromosome inactivation: advances in understanding facultative heterochromatin formation. Nat Rev Genet.

[5] Cedar H, Bergman Y (2012). Programming of DNA methylation patterns. Annu Rev Biochem..

[6] Feinberg AP (2007). Phenotypic plasticity and the epigenetics of human disease. Nature.

[7] Cancer Genome Atlas Research N (2011). Integrated genomic analyses of ovarian carcinoma. Nature.

[8] Cancer Genome Atlas N (2012). Comprehensive molecular portraits of human breast tumours. Nature.

[9] Hudson TJ, Anderson W, Artez A, Barker AD, Bell C, International Cancer Genome C (2010). International network of cancer genome projects. Nature.

[10] Laird PW (2010). Principles and challenges of genome-wide DNA methylation analysis. Nat Rev Genet.

[11] Bourgon R, Lu S, Yan Y, Lackner MR, Wang W, Weigman V et al. High-throughput detection of clinically relevant mutations in archived tumor samples by multiplexed PCR and next-generation sequencing. Clin Cancer Res. 2014. doi:10.1158/1078-0432.CCR-13-3114. 10.1158/1078-0432.CCR-13-3114 24573554

[12] Paliwal A, Temkin AM, Kerkel K, Yale A, Yotova I, Drost N (2013). Comparative anatomy of chromosomal domains with imprinted and non-imprinted allele-specific DNA methylation. PLoS Genet.

[13] Warnecke PM, Stirzaker C, Melki JR, Millar DS, Paul CL, Clark SJ (1997). Detection and measurement of PCR bias in quantitative methylation analysis of bisulphite-treated DNA. Nucleic Acids Res.

[14] Wojdacz TK, Hansen LL, Dobrovic A (2008). A new approach to primer design for the control of PCR bias in methylation studies. BMC Res Notes..

[15] Clark SJ, Statham A, Stirzaker C, Molloy PL, Frommer M (2006). DNA methylation: bisulphite modification and analysis. Nat Protoc.

[16] Ralser M, Querfurth R, Warnatz HJ, Lehrach H, Yaspo ML, Krobitsch S (2006). An efficient and economic enhancer mix for PCR. Biochem Biophys Res Commun.

[17] Krueger F, Andrews SR (2011). Bismark: a flexible aligner and methylation caller for Bisulfite-Seq applications. Bioinformatics.

[18] Langmead B, Salzberg SL (2012). Fast gapped-read alignment with Bowtie 2. Nat Methods.

