# Supplementary figures and images for: Multiplex bisulfite PCR resequencing of clinical FFPE DNA
Source: Clin Epigenetics. 2015 Mar 17;7(1):28. doi: 10.1186/s13148-015-0067-3 (PMC4389706; doi:10.1186/s13148-015-0067-3)

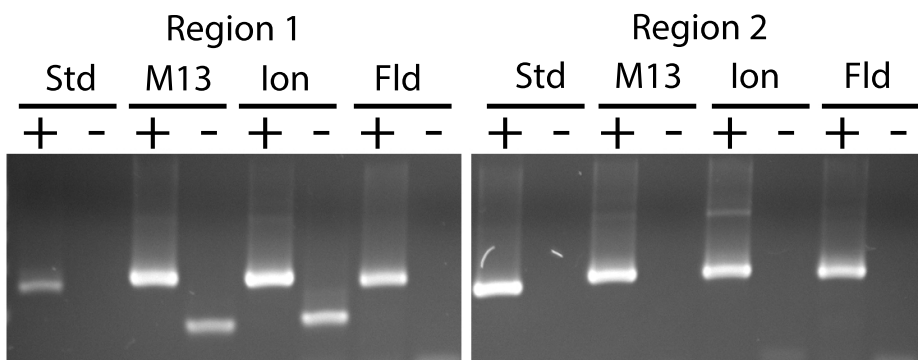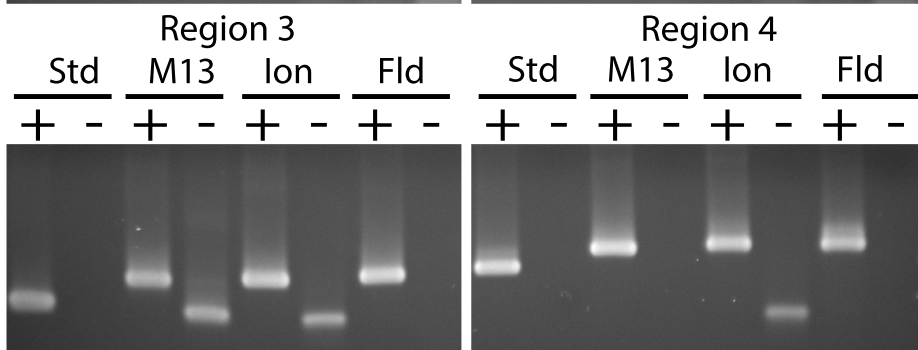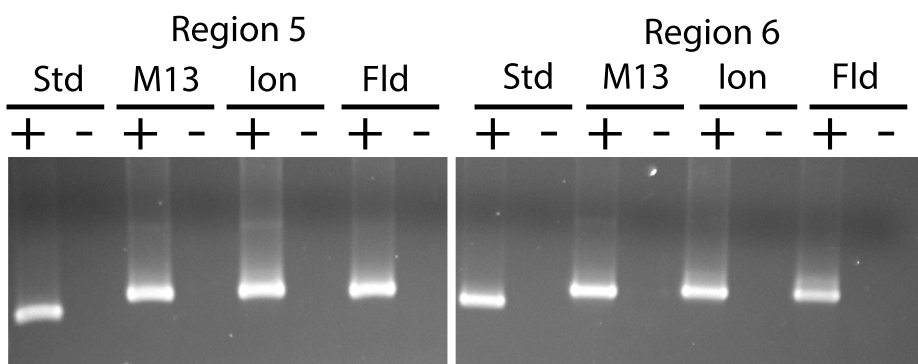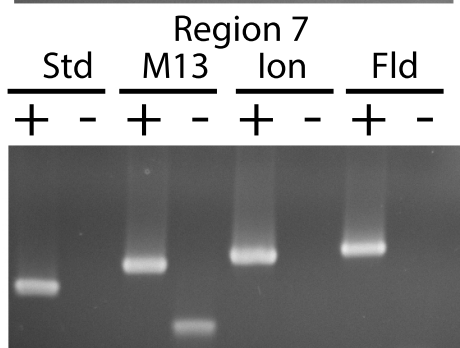

Supplement: Additional file 1: Figure S1. — To assess the performance of different fusion sequences in targeted resequencing, seven different pairs of bisulfite PCR primers were evaluated with and without additional sequences added to their 5' end. (−) = No template control; Std = primers with no fusion sequence; M13 = primers with M13 sequences; Ion = primers with Ion Torrent P and A1 sequences; Fld = Fluidigm’s CS sequences. Although all primer sets performed well under stringent conditions, only Fluidigm’s CS sequences gave no observable dimer product in the negative control. [file 13148_2015_67_MOESM1_ESM.pdf]

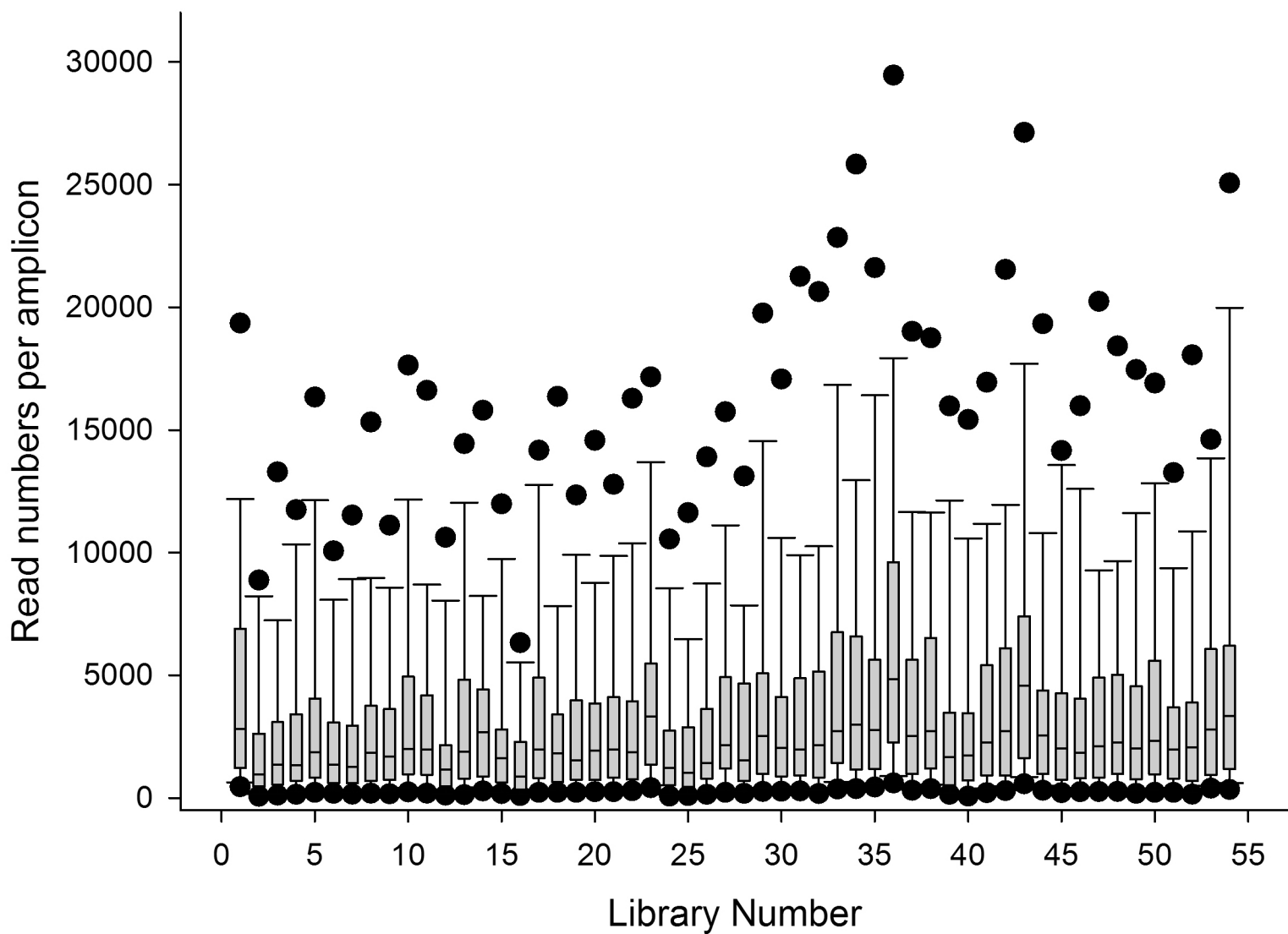

Supplement: Additional file 3: Figure S3. — The distribution of read counts for 56 bisulfite DNA amplicons across 54 libraries. Although the spread in read numbers between the lowest and highest amplicons for each library is less than two orders of magnitude (that is, 200 to 20,000), the majority of amplicons have read counts which cluster within one order of magnitude of each other. Whiskers: 10th to 90th percentiles; black circles: 5th and 95th percentiles. [file 13148_2015_67_MOESM3_ESM.pdf]

# Methylated Controls

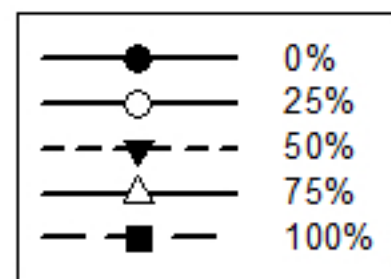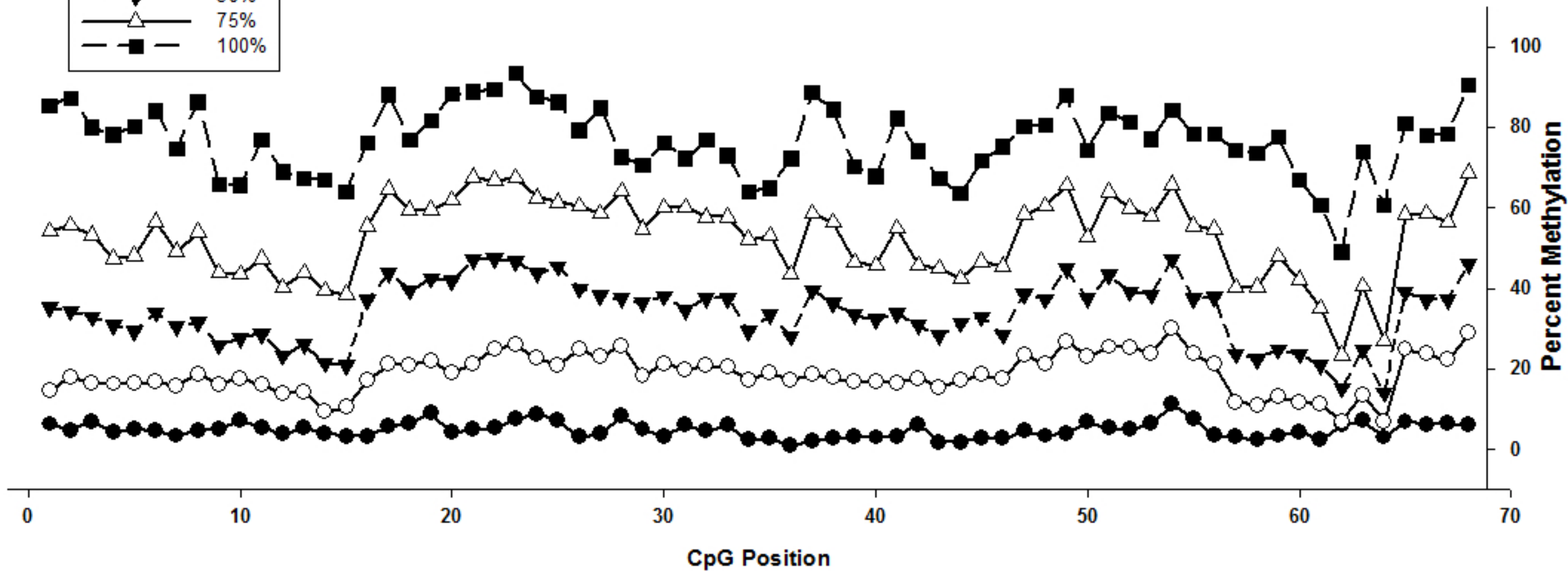

Supplement: Additional file 4: Figure S4. — Representative methylation bias plot for 68 CpGs across 8 amplicons in the library. Although particular CpG positions are observed to deviate from the median value (for example, CpG 62 and 64), overall, the assay is able to distinguish between different methylation percentages across every CpG. [file 13148_2015_67_MOESM4_ESM.pdf]
