# Supplementary material for: Multiplex bisulfite PCR resequencing of clinical FFPE DNA
Source: Clin Epigenetics. 2015 Mar 17;7(1):28. doi: 10.1186/s13148-015-0067-3 (PMC4389706; doi:10.1186/s13148-015-0067-3)
Supplement: Additional file 2: Figure S2. — Thirty gDNA primer pairs were divided equally into six pools and amplified concurrently at four different primer concentrations. Each pool was then sequenced on either an Ion Torrent (A) or MiSeq (B) to assess individual amplicon proportion, as well as whether particular sequencing platforms alter amplicon proportionality. The Y-axis represents the percentage proportion for each amplicon, as a total of its particular multiplex pool. Whiskers: 10th to 90th percentiles; black circles: outliers. [file 13148_2015_67_MOESM2_ESM.pdf]

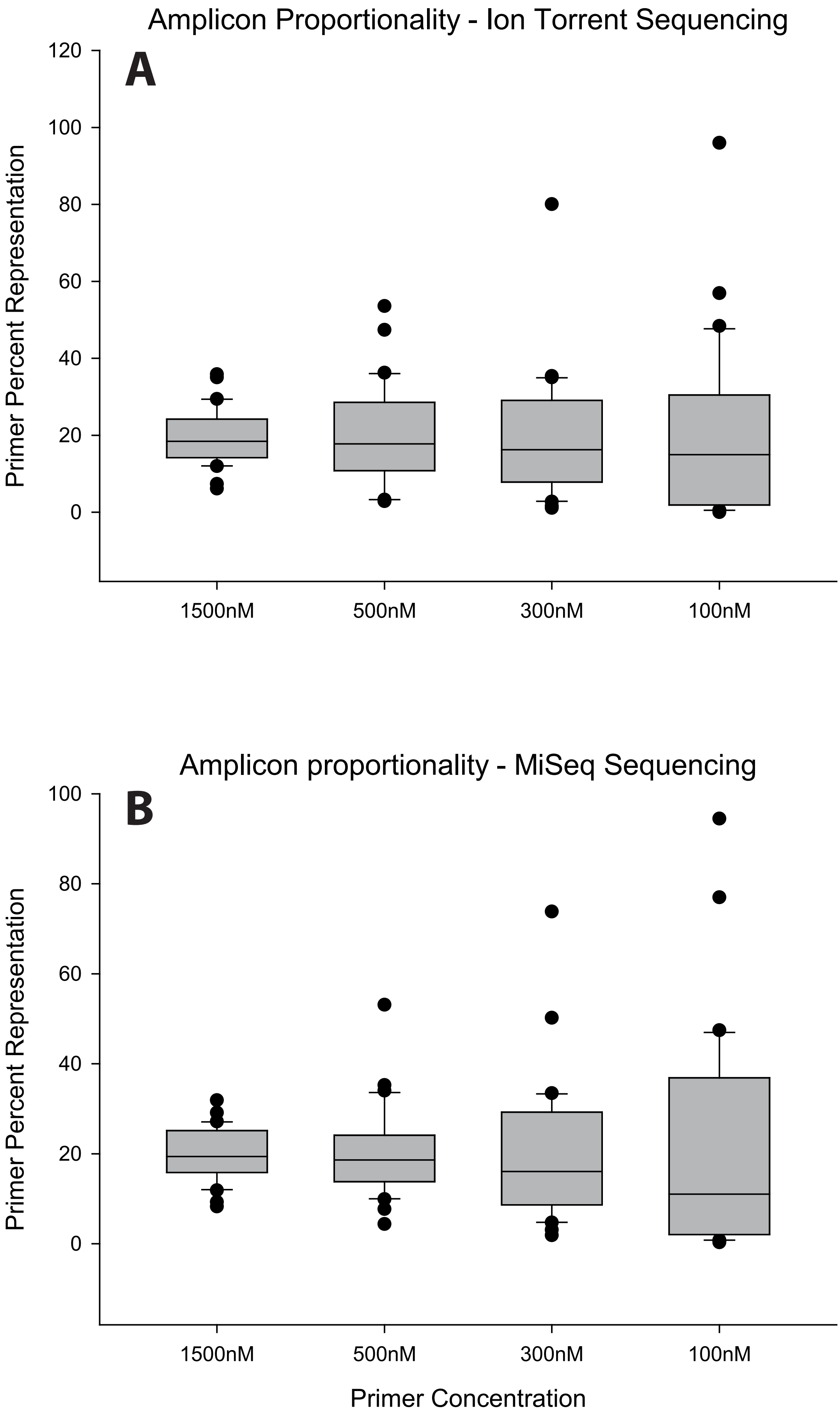
Amplicon Proportionality - Ion Torrent Sequencing
A
Primer Percent Representation
120
100
80
60
40
20
0
1500nM
500nM
300nM
100nM
Amplicon proportionality - MiSeq Sequencing
B
Primer Percent Representation
100
80
60
40
20
0
1500nM
500nM
300nM
100nM
Primer Concentration
