# Supplementary material for: Multiplex bisulfite PCR resequencing of clinical FFPE DNA
Source: Clin Epigenetics. 2015 Mar 17;7(1):28. doi: 10.1186/s13148-015-0067-3 (PMC4389706; doi:10.1186/s13148-015-0067-3)
Supplement: Additional file 5: Table S1. — Spread of standard deviation methylation values across Watson strand amplicons, based on three technical replicates. The maximal standard deviation observed in this dataset was due to a single amplicon at low coverage; removing this amplicon reduced the maximum standard deviation observed for a single CpG from 23.8% to 8.04%. [file 13148_2015_67_MOESM5_ESM.doc]

**Additional file 5: Table S1**. Spread of standard deviation methylation values across Watson-strand amplicons, based on three technical replicates. The maximal standard deviation observed in this dataset was due to a single amplicon at low coverage; removing this amplicon reduced the maximum standard deviation observed for a single CpG from 23.8% to 8.04%.

|  | 293 CpGs  33 amplicons | 281 CpGs  32 amplicons |
| --- | --- | --- |
| Number of Amplicons | 33 | 32 |
| Number of CpGs | 293 | 281 |
| Minimum SD | 0.0043 | 0.0043 |
| Maximum SD | 23.8097 | 8.0415 |
| Median SD | 1.2152 | 1.2043 |
| Mean SD  Range (Min SD – Max SD) | 1.5450  23.8054 | 1.4261  7.9985 |
|  |  |  |
