# Supplementary material for: Multiplex bisulfite PCR resequencing of clinical FFPE DNA
Source: Clin Epigenetics. 2015 Mar 17;7(1):28. doi: 10.1186/s13148-015-0067-3 (PMC4389706; doi:10.1186/s13148-015-0067-3)
Supplement: Additional file 6: Table S2. — Standard deviation values for tumour samples 1 to 13 across three technical replicates for one representative amplicon (N.B. Only samples 1 to 4 are shown in Figure 5). The standard deviation represents the value by which the technical replicates for each sample deviate from the mean percent methylation value. [file 13148_2015_67_MOESM6_ESM.doc]

**Additional file 6: Table S2**. Standard Deviation values for tumour samples 1 - 13 across three technical replicates for one representative amplicon (**N.B.** Only samples 1 – 4 are shown in **Figure 5**). The standard deviation represents the value by which the technical replicates for each sample deviate from the mean percent-methylation value.

|  | **CpG Position (+/− %)** | | | | | | | | | | | | |
| --- | --- | --- | --- | --- | --- | --- | --- | --- | --- | --- | --- | --- | --- |
|  | 1 | 2 | 3 | 4 | 5 | 6 | 7 | 8 | 9 | 10 | 11 | 12 | 13 |
| Sample 1 | 0.47 | 1.84 | 1.87 | 0.69 | 0.32 | 1.71 | 1.21 | 1.62 | 1.07 | 1.11 | 1.96 | 0.53 | 0.77 |
| Sample 2 | 1.39 | 1.29 | 2.29 | 0.68 | 2.15 | 2.84 | 2.36 | 1.88 | 0.78 | 0.60 | 1.01 | 1.37 | 1.33 |
| Sample 3 | 0.99 | 1.32 | 1.26 | 0.43 | 1.59 | 2.02 | 2.20 | 2.19 | 0.62 | 0.34 | 0.44 | 0.33 | 1.42 |
| Sample 4 | 0.97 | 1.88 | 2.10 | 0.23 | 1.08 | 1.92 | 0.76 | 1.27 | 0.58 | 0.27 | 0.13 | 2.87 | 1.36 |
| Sample 5 | 5.30 | 4.18 | 5.88 | 5.84 | 1.75 | 1.90 | 3.09 | 1.33 | 1.46 | 0.33 | 0.69 | 1.59 | 1.05 |
| Sample 6 | 2.74 | 1.98 | 0.58 | 1.50 | 2.07 | 2.24 | 0.67 | 1.36 | 0.68 | 1.05 | 1.16 | 0.36 | 3.37 |
| Sample 7 | 0.55 | 2.57 | 2.05 | 1.77 | 0.89 | 1.51 | 2.05 | 2.56 | 0.49 | 0.40 | 0.60 | 1.18 | 3.59 |
| Sample 8 | 2.86 | 1.26 | 2.09 | 2.50 | 1.70 | 1.71 | 1.49 | 1.74 | 0.66 | 0.41 | 0.47 | 1.83 | 0.73 |
| Sample 9 | 2.12 | 0.98 | 2.37 | 2.05 | 1.32 | 2.46 | 1.01 | 0.59 | 0.78 | 0.27 | 0.85 | 1.42 | 0.41 |
| Sample 10 | 1.49 | 0.58 | 0.76 | 1.22 | 0.86 | 0.61 | 2.42 | 1.05 | 0.15 | 0.59 | 0.85 | 1.03 | 1.06 |
| Sample 11 | 1.66 | 2.97 | 2.15 | 0.23 | 0.89 | 1.46 | 1.93 | 2.17 | 2.05 | 2.78 | 3.41 | 1.71 | 1.26 |
| Sample 12 | 1.07 | 2.14 | 1.27 | 1.34 | 1.38 | 1.67 | 2.23 | 0.79 | 1.27 | 0.41 | 0.71 | 0.75 | 2.43 |
| Sample 13 | 2.19 | 0.64 | 3.43 | 1.83 | 1.74 | 3.40 | 1.01 | 0.72 | 0.55 | 1.74 | 0.82 | 2.76 | 3.81 |
